# Flux-sum coupling analysis of metabolic network models

**DOI:** 10.1371/journal.pcbi.1012972

**Published:** 2025-04-07

**Authors:** Mihriban Seyis, Zahra Razaghi-Moghadam, Zoran Nikoloski

**Affiliations:** 1 Bioinformatics Department, Institute of Biochemistry and Biology, University of Potsdam, Potsdam, Germany; 2 Systems Biology and Mathematical Modeling Group, Max Planck Institute of Molecular Plant Physiology, Potsdam, Germany; Ecole Polytechnique Fédérale de Lausanne, SWITZERLAND

## Abstract

Metabolites acting as substrates and regulators of all biochemical reactions play an important role in maintaining the functionality of cellular metabolism. Despite advances in the constraint-based framework for genome-scale metabolic modeling, we lack reliable proxies for metabolite concentrations that can be efficiently determined and that allow us to investigate the relationship between metabolite concentrations in specific metabolic states in the absence of measurements. Here, we introduce a constraint-based approach, the flux-sum coupling analysis (FSCA), which facilitates the study of the interdependencies between metabolite concentrations by determining coupling relationships based on the flux-sum of metabolites. Application of FSCA on metabolic models of *Escherichia coli*, *Saccharomyces cerevisiae*, and *Arabidopsis thaliana* showed that the three coupling relationships are present in all models and pinpointed similarities in coupled metabolite pairs. Using the available concentration measurements of *E. coli* metabolites, we demonstrated that the coupling relationships identified by FSCA can capture the qualitative associations between metabolite concentrations and that flux-sum is a reliable proxy for metabolite concentration. Therefore, FSCA provides a novel tool for exploring and understanding the intricate interdependencies between the metabolite concentrations, advancing the understanding of metabolic regulation, and improving flux-centered systems biology approaches.

## Author summary

Metabolites are the building blocks and regulators of all biochemical reactions, making them essential for the maintenance of life. Understanding how metabolite concentrations change and interact is crucial to the study of metabolism, but measuring them directly is often difficult. To address this challenge, we have developed Flux-Sum Coupling Analysis (FSCA), a new computational approach that uncovers relationships between metabolite concentrations using mathematical models of metabolism. By applying FSCA to models of bacteria (*Escherichia coli*), yeast (*Saccharomyces cerevisiae*), and plants (*Arabidopsis thaliana*), we discovered consistent patterns in how metabolites are coupled. We also tested our approach on real metabolite data from *E. coli* and found that FSCA successfully captured key relationships between metabolite concentrations. This suggests that flux sum, a property of metabolic networks, can serve as a useful predictor of metabolite levels. Our findings provide a new way to study metabolism without the need for extensive experimental data.

## Introduction

Constraint-based modeling of metabolism has resulted in a toolbox of computational approaches that facilitate the prediction of complex phenotypes [1], the design of metabolic engineering strategies [2], as well as the discovery of functionally relevant dependences between reaction fluxes [3]. With the exception of thermodynamic metabolic flux analysis [4], all approaches in the constraint-based modeling framework focus solely on reaction fluxes and remain silent on the prediction of metabolite concentrations and dependencies between them. Expanding the applicability of the constraint-based modeling framework to study metabolite concentrations can help in the identification of determinants of metabolic functionalities.

The stoichiometry of metabolic reactions allows the identification of conservation relationships of metabolite concentrations, i.e. a subset of metabolites whose total concentration is invariant in any state of the system (i.e. steady state and transients) [5]. These relationships already indicate that for a fixed conserved pool, an increase in the concentration of one metabolite is bound to decrease the concentration of at least one metabolite in the conservation relation [6]. Inspired by flux coupling analysis (FCA) [7], which identifies relations between fluxes of reactions at steady-state, Nikolaev et al. (2005) proposed metabolite concentration coupling analysis (MCCA). This constraint-based approach categorizes (ordered) pairs of metabolites as fully, partially, and directionally coupled based on their participation in conservation relationships of concentrations.

In an attempt to render reaction-centric constraint-based modeling applicable to the study of metabolite concentrations, Kim et al. (2007) proposed the flux-sum analysis [8]. The flux-sum of a metabolite denotes the total flux affecting the pool of the metabolite and can be determined solely from the stoichiometry of the network using linear programming in the context of constraint-based modeling. Provided that enzyme levels are maintained constant, a larger flux through an irreversible reaction can, in the simplest scenario, be attributed to increasing the concentration of at least one of the metabolites serving as substrates. Therefore, flux-sum analysis provides metabolite-centric predictions and allows comparisons of metabolic states. However, there has been no assessment of the extent to which flux-sums reflect metabolite concentrations [9]. In addition, there have been no attempts to examine if the association between metabolite concentrations, often performed in the context of metabolic correlation network analysis, is reflected in the dependence between flux-sums for pairs of metabolites [10].

Here we present the flux-sum coupling analysis (FSCA) that builds on the concept of flux-sum and FCA to categorize pairs of metabolites based on the relations between their flux-sums. Our analyses indicate that the defined coupling relations based on the flux-sum of metabolites are present in different metabolic models. In addition, we investigate if flux-sums and their couplings reflect concentrations of metabolites and their linear relationships. We note that FSCA is particularly useful to study the relationships between metabolites, especially when it is difficult to experimentally measure metabolite concentrations. By integrating these analyses, we aim to establish flux-sum as a biologically meaningful quantity for understanding metabolite concentrations and their dependencies in metabolic networks.

## Results and discussion

### Coupling of metabolite flux-sums

Given that flux-sum has been proposed as a reliable estimate of metabolite concentration [11], and inspired by the concept of flux coupling [7], here we introduce flux-sum coupling analysis (FSCA). For the metabolite $m_{i}$ , the flux-sum, $\phi_{m_{i}}$, is defined as the sum of fluxes through the metabolite, weighted by the absolute value of the stoichiometric coefficients. It can be expressed mathematically as $\phi_{m_{i}}=\left|N_{m_{i},:}\right|\cdot v$, where, $N_{m_{i},:}$ represents the *i*^th^ row of the stoichiometric matrix $N,\left|\cdot \right|$, denotes the absolute value of this row, and *v* is a flux vector, i.e. a vector containing a flux distribution.

**Fig 1:**
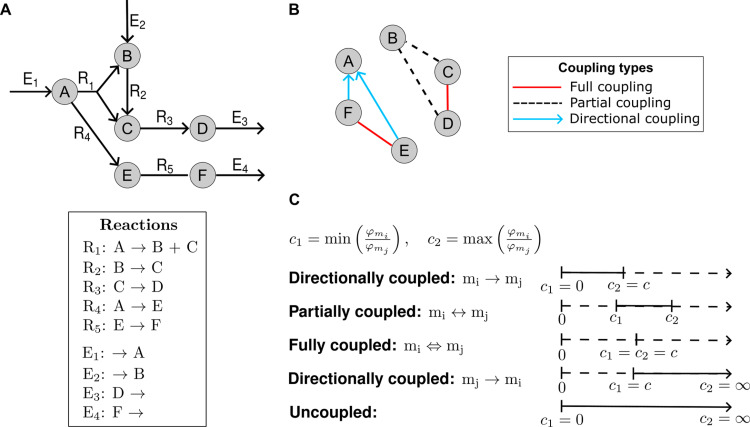
Illustration of the flux-sum coupling concept. A. A toy network representation consisting of six metabolites, denoted by A - G, and nine reactions, depicted in the box. B. Illustration of flux-sum couplings on the toy network. Nodes represent metabolites A-F, and labeled edges represent coupling relations (see legend). C. Classification of flux-sum couplings of metabolites iand *j*. Flux-sums of metabolites *i* and *j* are denoted as $\Phi m_{i}$ and $\Phi m_{j}$, respectively. The coupling classes are assigned based on the minimum and maximum flux-sum ratios, $c_{1}$ and $c_{2}$, which can be achieved at steady state.

Under steady-state constraints, if a non-zero flux-sum of one metabolite implies a non-zero flux-sum of another, the two metabolites are said to be coupled. This definition can be further refined to distinguish three types of coupling for metabolites $m_{i}$ and $m_{j}$ based on their respective flux-sums, $\phi_{m_{i}}$ and $\phi_{m_{j}}$. Inspired by the definition of coupling for reaction fluxes, metabolites $m_{i}$ and $m_{j}$ can be in three coupling relations which we illustrate using a toy network of six metabolites interchanged by five internal, $R_{1}$–$R_{5}$, and four exchanges, $E_{1}$-$E_{4}$, reactions (Fig 1A): (1) *directionally coupled*, if a non-zero flux-sum for $m_{i}$ implies a non-zero flux-sum for $m_{j}$, but not *vice versa*; for instance, metabolites E and F are directionally coupled to A (Fig 1B). (2) *partially coupl*ed, if a non-zero flux-sum for $m_{i}$ implies a non-zero flux-sum for $m_{j}$ and *vice versa*; for instance, B is ppartiallycoupled with C and D (Fig 1B) and (3) fully coupled, when a non-zero flux-sum for $m_{i}$ not only implies a non-zero but also a fixed flux-sum for $m_{j}$ and *vice versa*, as is the case for metabolites F and E as well as C and D (Fig 1B). Metabolite pairs not falling into any of these three categories are considered uncoupled.

Identifying the three coupling types can be performed by solving two linear fractional programming problems (see Methods), resulting in the minimum and the maximum values, $c_{1}$ and $c_{2}$, for the ratio $\frac{\phi_{m_{i}}}{\phi_{m_{j}}}=\frac{\left|N_{m_{i},:}\right|\times v}{\left|N_{m_{j},:}\right|\times v}$. The first case, where $c_{1}$ equals zero and $c_{2}$ is a finite constant, which implies that ϕmj≥ϕmic2 and the two metabolites are directionallycoupled, denoted by $m_{i}\to m_{j}$. Similarly, if $c_{1}$ is a finite constant and $c_{2}$ is unbounded, then $\phi_{m_{i}}\geq c_{1}\cdot \phi_{m_{j}}$, indicating a directional coupling from $m_{j}$ to $m_{i}$ ($m_{j}\to m_{i}$). Two metabolites are partially coupled, denoted by $m_{i}↔m_{j}$, if both $c_{1}$ and $c_{2}$ are finite but unequal constants. Full coupling ($m_{i}\Leftrightarrow m_{j}$) occurs when $c_{1}=c_{2}$ and they are finite. Finally, the two metabolites are uncoupled if $c_{1}$ is zero and $c_{2}$ is infinite (Fig 1C).

### Metabolites with coupled flux-sums are found across models of different organisms

**Fig 2:**
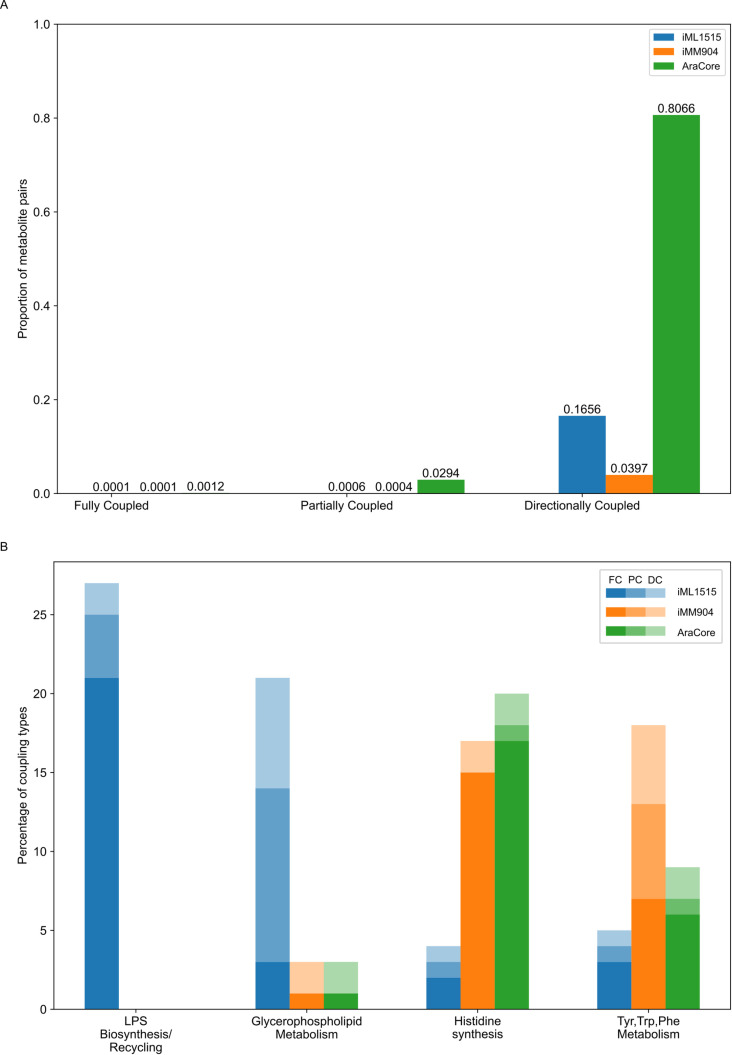
Flux-sum coupling and mutual metabolic pathway types across three metabolic models. Models of three organisms were used to compare the fraction of particular flux-sum coupling types, namely the iML1515 model of *E. coli*, the iMM904 model of *S. cerevisiae*, and the AraCore model of *A. thaliana*. A. Fraction of pairs that belong to the particular flux-sum coupling type, namely: fully, partially, directionally coupled metabolites in the three metabolic models. B. Representation of particular flux-sum couplings in selected metabolic pathways. This bar chart illustrates the percentage of coupling types in selected metabolic pathways across the three metabolic models. The fully, partially, and directionally coupled pairs were found to be most related to Lipopolysaccharide Biosynthesis/Recycling, Glycerophospholipid Metabolism, Histidine Synthesis, and Tyrosine, Tryptophan, and Phenylalanine Metabolism. Within each model, the coupling types are represented with hues, from darkest to lightest: full coupling (FC), partial coupling (PC), and directional coupling. (DC) (see legend).

Next, we applied FSCA to identify the metabolite pairs with coupled flux-sums in the metabolic networks of *E. coli* (iML1515), *S. cerevisiae* (iMM904), and *A. thaliana* (AraCore) (see Methods). In the *E. coli* iML1515 model, 0.007% of the metabolite pairs showed full coupling, 0.063% showed partial coupling, and 16.56% showed directional coupling (Fig 2A). For the *S. cerevisiae* iMM904 model, 0.010% of metabolite pairs were fully coupled, 0.036% partially coupled, and 3.97% were directionally coupled. Similarly, in the AraCore model, we found that 0.12% of metabolite pairs were fully coupled, 2.94% were partially coupled, and 80.66% were directionally coupled (see Fig 2A). Of the three coupling types, directionally coupled pairs are the most common in the three analyzed models. The prevalence of directionally coupled metabolite pairs is due to the definition of directional coupling, which allows for more pairs to meet the criteria. In contrast, full coupling is the least common due to its more restrictive definition, which imposes strict conditions for metabolite pairs to be considered fully coupled. These findings illustrate the variability of coupling types in different metabolic models that reflect the differences in the structural properties of these networks.

To further explore the variability of coupling types across metabolic models, we analyzed the metabolites most frequently involved in the defined couplings. Our analysis revealed that the top ten metabolites associated with coupling interactions were unique to each model, with no overlap observed among the studied models (see S3 Table). This uniqueness underscores the specificity of coupling patterns within individual metabolic networks and highlights the influence of model-specific features as well as the species-specific flux distributions in shaping metabolic relationships.

Furthermore, we investigated the correlation between the frequency of a metabolite's involvement in coupling interactions and the number of reactions it participates in within the metabolic network. The correlation values were -0.20 for the *E. coli* iML1515 model, 0.05 for the *S. cerevisiae* iMM904 model, and -0.17 for the AraCore model. The consistently low correlations observed across all three models suggest that a metabolite's involvement in coupling interactions is largely independent of its overall connectivity within the network. Therefore, given these results, we conclude that the flux-sum is a functional property that reflects both the structure of the model and underlying flux distributions supported.

Overall, in the three studied models, the metabolites involved in identified couplings are predominantly associated with lipopolysaccharide biosynthesis/recycling, glycerophospholipid metabolism, histidine synthesis and the metabolism of tyrosine, tryptophan and phenylalanine. The distribution of coupled metabolite pairs in these pathways varies between models (see Fig 2B). In the metabolic models of *E. coli*

the coupled metabolite pairs are predominantly associated with glycerophospholipid metabolism as well as transport, outer membrane porin pathway. In contrast, in the AraCore and *S. cerevisiae* models, they are predominantly associated with histidine synthesis. In the *E. coli* iML1515 model, 20.55% of fully coupled pairs were associated with lipopolysaccharide biosynthesis/recycling, representing the highest percentage of fully coupled pairs associated with a specific pathway in this model. The highest percentage of fully coupled pairs linked to a specific pathway is 14.89% in the *S. cerevisiae* iMM904 model and 17.11% in the AraCore model, both associated with histidine synthesis.

**Fig 3:**
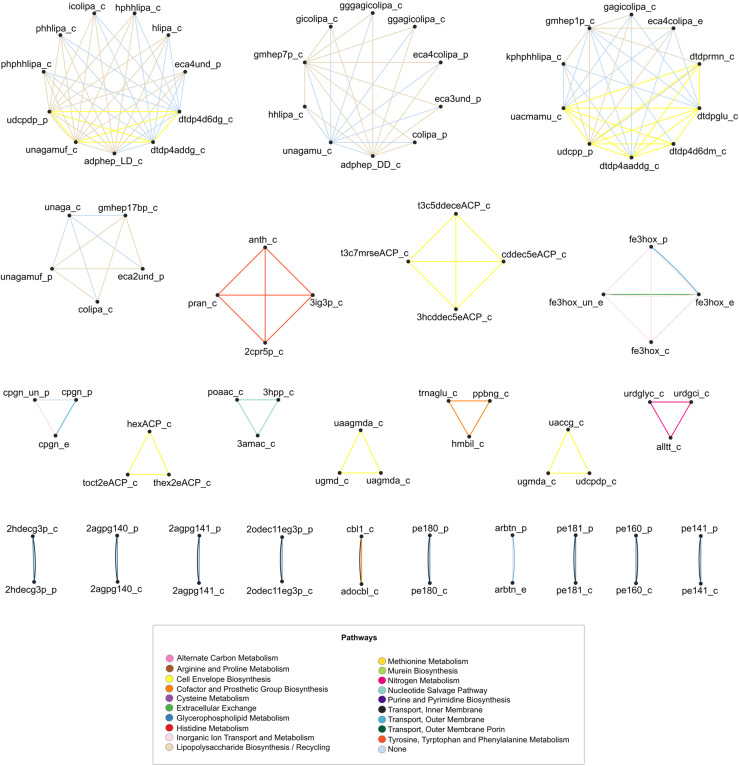
Network representation of metabolites with fully coupled flux-sums in the iML1515 model. Nodes represent metabolites, and edges depict the mutual pathways in which pairs of metabolites participate. Nodes with a degree smaller than 1 have been removed from the graph to improve visualization. Abbreviations for metabolites are used in the figure. The full names of the metabolites are given in Table A in S2 Table.

By definition, full coupling results in equivalence classes, which form modules of metabolites with fully coupled flux-sums. For instance, the *E. coli* iML1515 model results in 24 modules of metabolites with fully coupled flux-sums, with module sizes ranging from 2 to 11 metabolites (see Fig 3). In the network representation of these fully coupled classes, nodes represent metabolites connected by edge(s) when they share pathway(s). The largest class contains 11 metabolites, belonging to the cytosol and periplasmic compartments and are involved in lipopolysaccharide biosynthesis/recycling and cell envelope biosynthesis (S4 Table). In the network representation in Fig 3, where all edges within an equivalence class are shown in a single colour, it is clear that metabolites within that class are not only fully coupled, but also share the same pathway. The largest class of this type contains four different metabolites in the cytosol compartment, namely, Anthranilate, N-(5-Phospho-D-ribosyl)anthranilate, 1-(2-Carboxyphenylamino)-1-deoxy-D-ribulose5-phosphate, and C'-(3-Indolyl)-glycerol3-phosphate, which participate in the ‘Tyrosine, Tryptophan, and Phenylalanine Metabolism’ pathway. The largest classes in the network representations of the fully coupled classes for *S. cerevisiae* and *A. thaliana* contain 8 and 10 metabolites, respectively (S1 and S2 Figs, and S5 and S6 Tables). The metabolites in the largest classes of the *S. cerevisiae* and *A. thaliana* models all share the same pathway, which is the histidine sythesis in both models.

The Metabolite Coupling Analysis (MCA) approach [12] has a similar formulation to the FSCA method. However, there are important differences in the techniques used to solve these problems. While MCA relies on Mixed Integer Linear Programming (MILP), FSCA uses the Charnes-Cooper transformation [13], which reformulates the problem in a Linear Programming (LP) framework (see Methods). To directly compare these methods, we applied FSCA to the same *E.coli* iJO1366 metabolic model [14] used in the MCA study. Notably, MCA reported a total of 666,435 metabolite pairs, whereas the iJO1366 model contains 1,805 metabolites, resulting in 3.256.220 possible unique pairs. This discrepancy suggests that additional constraints were applied in MCA that were not explicitly detailed in the referenced publication. As the MCA study only reported the number of metabolite pairs within each category of fully coupled (FC), partially coupled (PC), directionally coupled (DC), and uncoupled (UC) pairs, our comparison is necessarily limited to the number of pairs in each category. The FSCA method identified 257 pairs in the FC class, 2311 pairs in the PC class, and 555831 pairs in the DC class. MCA, on the other hand, reported 1237 FC pairs, 114 PC pairs, and 24789 DC pairs. The differences in these numbers highlight that the methodological choices influence the classification and quantification of metabolite couplings.

### Concentration dependencies do not reflect the degree of metabolite coupling

FSCA aims to uncover dependencies between linear combinations of fluxes that shape the concentration of specific metabolites. Here we investigated the extent to which the degree of coupling reflects the correlations between metabolite concentrations. To examine this association, we used the available concentration measurements of *E. coli*’s metabolites. The concentration measurements were gathered from the study of Ishii et al. (2007), where multiple high-throughput measurements were conducted to study the response of *E. coli* cells to 22 genetic and five environmental perturbations [15]. The genetic perturbations resulted from 22 gene knockouts (see S1 Table) and the cells were grown in a glucose-limiting chemostat. The environmental perturbations were studied at different dilution rates (0.1, 0.2, 0.4, 0.5, and 0.7) in chemostat cultures, where the glucose concentration was controlled and varied from an almost glucose-starved state to a non-limiting supply.

**Fig 4:**
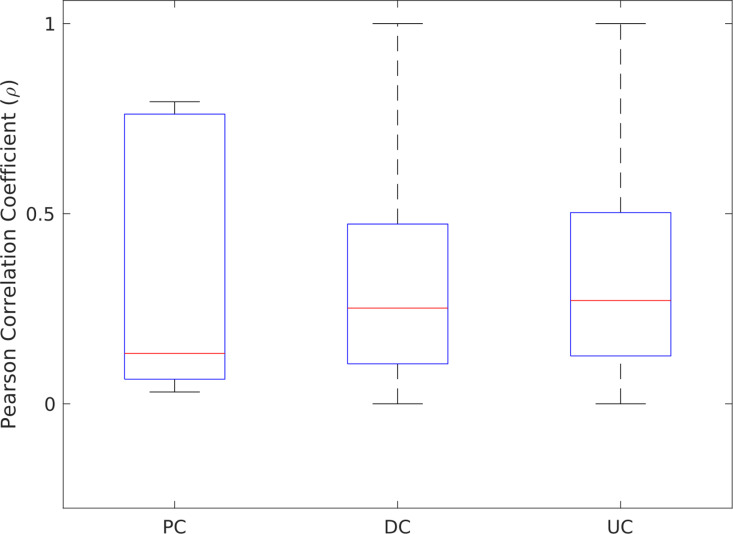
Distribution of correlations of measured metabolite levels across flux-sum coupling types in *E. coli*. Boxplots comparing Pearson correlations of measured metabolite levels for fully coupled (FC), partially coupled (PC), directionally coupled (DC), and uncoupled (UC) pairs in the iML1515 model of *E. coli.*

Metabolite pairs in the iML1515 model with available concentration measurements were categorized into FC, PC, DC and UC groups. Altogether, there are 10 metabolite pairs in the FC group, 20 metabolite pairs in the PC group, 15990 metabolite pairs in the DC group, and 1011 metabolite pairs in the UC group, for which the concentration measurements were available. However, some pairs included the same metabolite but from different compartments. As the measured metabolite concentrations are not compartment-specific, these pairs were excluded from the analysis, leaving no pairs in the FC group, 12 in the PC group, 15905 in the DC group, and 1006 in the UC group. The absolute Pearson correlations were calculated for the concentrations of metabolite pairs within each group. The PC, DC and UC groups have average absolute Pearson correlation coefficients of 0.33, 0.32, and 0.33, respectively. Initially, we expected the FC group to have the highest average correlation, followed by the PC group, with the DC and UC groups having lower correlations. However, as no pairs remained in the FC group, we could not assess its correlation. In addition, the average correlation in the PC group was similar to that of the DC and UC groups, contrary to what we expected (Fig 4). This could, in part, be due to the lack of compartment-specific concentration measurements as well as the small number of pairs in the PC group, which may have limited the ability to detect distinct dependency patterns. For the DC and UC groups, the observed low correlations are consistent with the fact that directional coupling and uncoupling describe relationships that are not symmetric or stoichiometrically tight. Specifically, in the DC group, the non-zero flux sum of one metabolite reflects the non-zero flux sum of the other, but this dependence does not require a proportional relationship in their concentrations. This finding is consistent with that of [16] which showed that fully coupled reaction pairs have, on average, higher transcription factor binding similarities than both directional and uncoupled ones.

### Metabolite flux-sum values can serve as a surrogate for metabolite concentrations

Given the expectation that the degree of coupling captures the correlation between concentration of metabolites, we further investigated whether the metabolite flux-sum itself can serve as a surrogate for metabolite concentration. This investigation was motivated by the hypothesis that if flux-sum coupling were to reflect dependencies between metabolite concentrations, then flux-sum itself might also carry meaningful information about individual metabolite concentrations, although this remains uncertain given the unexpected findings, above. To test this idea, we first compared available concentration measurements for *E. coli* metabolites [15] with the flux-sums of the corresponding metabolites. The parsimonious flux balance analysis (pFBA) method was used to calculate the fluxes required for the flux-sum calculations. To assess the precision of the resulting flux values, a tailored variability analysis was carried out to determine the admissible ranges for each flux. The results showed that, on average, the logarithmic ratio of the maximum and the minimum flux values calculated at the minimum total flux ranges from 0 to 0.5. This indicates that the maximum and minimum flux values for each reaction are closely matched, which in turn ensures that the flux-sums are reliable and robust.

From the 258 metabolites with available concentration measurements, 114 were included in the *E. coli* iML1515 model. Notably, some metabolites in the model were found in multiple compartments, and their flux-sum were calculated as the total over these compartments. For the 114 metabolites, we calculated the Pearson correlations between the flux-sums and concentrations across the conditions for which measurements were available (S7 Table). We note that the Pearson correlation could not be calculated for 48 out of the 114 metabolites because the flux-sum variances were zero across the 27 conditions due to the usage of pFBA in calculation of the flux-sum (see Methods).

Of the remaining 66 metabolites, 18 showed significant Pearson correlations (p-values smaller than 0.05), in the range 0.38 to 0.77, mostly determined from experimental measurements for all 27 conditions (see S3 Fig). For instance, Adenosine Diphosphate (ADP), Alanine (Ala), Adenosine Monophosphate (AMP), Arginine (Arg), Aspartate (Asp), Carbamoylaspartate (cbasp), Deoxycytidine Triphosphate (dCTP), Glycine (Gly), Guanosine Monophosphate (GMP), Inosine Monophosphate (IMP), Methionine (Met), N-Acetylglutamate (acglu), Nicotinamide Adenine Dinucleotide Phosphate (NADP), O-Acetylserine (acser), Pyridoxal 5-phosphate (pydx5p), and Threonine (Thr) showed Pearson correlation larger or equal to 0.45, while S-Adenosyl-L-methionine (amet) and GTP showed Pearson correlation of 0.41 and 0.38, respectively. Given the moderate correlations, these findings indicated that metabolite flux-sums could potentially be used as a surrogate for concentration of selected metabolites that cover important pathways, including: Cofactor and Prosthetic Group Biosynthesis, Nucleotide Salvage Pathway, Extracellular exchange, Alternate Carbon Metabolism, Lipopolysaccharide Biosynthesis / Recycling, Transpor reactions, Arginine and Proline Metabolism. However, we would like to stress that these findings are affected by the usage of pFBA in the calculation of flux-sums for particular experimental scenario. The ultimate test for the relation between flux-sums and metabolite concentrations would require tailored paired fluxomics and quantitative metabolomics data sets which are currently lacking.

## Conclusions

This study introduces FSCA, a novel constraint-based approach for analysis of metabolic network analysis and for the study of the intricate relationships between metabolites. FSCA builds on the concepts of flux-sum and FCA to systematically classify pairs of metabolites based on the relationships between their flux-sums. This classification can categorize metabolite pairs in descending order of relatedness: from fully coupled, indicating the strongest relationship, to partially coupled and directionally coupled, to uncoupled, representing the weakest association. We note that while flux-sums are features of metabolites, they can be fully determined using flux-centered approaches, allowing facile calculation using the constraint-based modeling framework.

Applying FSCA to a metabolic model of *E. coli* resulted in varying degrees of coupling for metabolite pairs, which were validated using published metabolite concentration data. The validation showed that the relationships identified by flux-sum are not merely theoretical but have practical relevance, effectively capturing qualitative associations between metabolite concentrations and further confirming the utility of FSCA in metabolic network analysis. Building on this evidence, and supported by the same published data, our analysis showed that flux-sum can potentially act as a surrogate for metabolite concentration, expandingits applicability in situations where direct measurement of metabolite concentrations is challenging or infeasible. The validity of using flux-sum as a proxy for metabolite concentration is further supported by evidence from a recent study that successfully used flux-sum as a feature to predict metabolite-protein interactions [17]. This evidence not only reinforces our findings but also highlights the broader applicability of flux-sum in metabolic research.

Looking ahead, future research can aim to use metabolite-flux-sums in the context of enzyme kinetics, particularly in the context of enzyme-constrained models that are becoming available for many model species [18]. Since the metabolic functionality of these models is further restricted by constraints on the total protein pool, this may have important implications on metabolite flux-sums. In addition, the observation that flux-sums can be used to investigate relationships between metabolite concentrations provides the means to expand the usage of metabolite correlations to constrain the predictions of feasible flux distributions using flux-sums, as recently attempted [19]. Importantly, we note that the predictions of flux-sums and their relationships depend on the metabolic model used. Further, investigations of their relevance in serving as proxies for metabolite concentrations must incorporate paired fluxomics and quantitative metabolomics measurements at a genome-scale over diverse range of conditions and genetic perturbations, which are currently lacking.

## Methods

### Flux-sum coupling (FSC) of metabolites

To determine the flux-sum couplings for a pair of metabolites, we relied on linear fractional programming (LFP) to identify the maximum and minimum ratio of flux-sums (i.e., maxϕmiϕmj, minϕmiϕmj) of the two metabolites:


$$\begin{array}{l} \underset{v}{max\left(min\right)}\frac{\phi_{m_{i}}}{\phi_{m_{j}}}=\frac{\left|N_{m_{i},:}\right|\times v}{\left|N_{m_{j},:}\right|\times v}\\ s.t\\ N\times v=0,\\ 0\leq v\leq v_{max}. \end{array}$$

The Charnes-Cooper transformation [13] was applied to convert this LFP into the following equivalent LP:


$$\begin{array}{l} \underset{\text{v',}t}{\text{(max(min))}}\text{|}N_{\left(m_{i},:\right)}|\times v^{\prime }\\ s.t.\\ N\times v'\;=0,\\ 0\leq v'\leq v_{max}\times t,\\ t\geq 0,\\ \left|N_{m_{j},:}\right|\times v^{\prime }=1, \end{array}$$

where the variable transformation $v^{\prime }=v\cdot t$ is used. The calculated minimum and maximum flux-sum ratios are in turn used to identify the types of coupling between the two metabolites. The use of an LP formulation of the problem guarantees that the global optimal values for the flux-sum ratios is determined.

FSC analysis was conducted using the metabolic models of *E. coli* (iML1515) [20], with 1877 metabolites and 2712 reactions, *S. cerevisiae* (iMM904) [21], with 1226 metabolites and 1577 reactions, and *A. thaliana* (AraCore) [22], with 407 metabolites and 549 reactions. All models were converted to irreversible models before FSC analysis. This involved splitting reversible reactions into forward and reverse reactions, both carrying non-negative fluxes.

### Metabolite flux-sum

The iML1515 model of *E. coli* [20] was used to simulate the genetic and environmental perturbations used in the study of Ishii et al. (2007) and to calculate the flux-sum for the metabolites in the model [15]. We first converted the metabolic model into an irreversible model which we used in the follow-up analyses. Genetic perturbations were simulated through gene knockouts mapped to model reactions via gene-protein-reaction (GPR) rules. In all perturbation scenarios, biomass rates were set to the corresponding measured dilution rates (see S1 Table).

The pFBA approach was used to determine a representative flux distribution for each genetic perturbation, using the following optimization problem:


$$\begin{array}{l} \underset{v}{min}\underset{i}{\sum }v_{i}\\ s.t.\\ N\times v\;=\;0,\\ v_{bio}\;=\;\mu \\ v_{i}=\;0,\;i\;\in \;knockoutreactions\\ 0\leq v\leq v_{max} \end{array}$$

where *N* is the stoichiometric matrix, *v* denotes a flux distribution, $v_{i}$ represents the flux through the *i*^th^ reaction, and *μ* is a measured dilution rate. Having determined the fluxdistribution, the flux-sum for metabolite *m* can be calculated by $\phi_{m}=\frac{1}{2}\underset{i}{\sum }\left|N_{m,i}\right|.v_{i}$.

## Implementation

The proposed LP formulation of FSC analysis is implemented in MATLAB Version: 23.2.0.242891 (R2023b) and is available at https://github.com/mihribanseyis/FSC.

## Supporting information

S1 Fig Network representation of metabolites with fully coupled flux-sums in the iMM904 model. Nodes represent metabolites, and edges depict the mutual pathways in which pairs of metabolites participate. Nodes with a degree smaller than 1 have been removed from the graph to improve visualization. Abbreviations for metabolites are used in the figure. The full names of the metabolites are given in Table B in S2 Table.
(DOCX)

S2 Fig Network representation of metabolites with fully coupled flux-sums in the AraCore model. Nodes represent metabolites, and edges depict the mutual pathways in which pairs of metabolites participate. Nodes with a degree smaller than 1 have been removed from the graph to improve visualization. Abbreviations for metabolites are used in the figure. The full names of the metabolites are given in Table C in S2 Table.
(DOCX)

S3 Fig Scatterplots of correlation between estimated flux-sums and measured metabolite levels. Scatter plots showing the flux sums of metabolites predicted by and relative metabolite concentrations over 27 conditions. The plots also include the Pearson correlation coefficients, along with p-values, and respective linear fits.
(DOCX)

S1 Table pFBA simulated conditions. (XLSX)

S2 Table (a) Metabolite abbreviations and names of fully coupled metabolite pairs for the iML1515 model, (b) Metabolite abbreviations and names of fully coupled metabolite pairs for the iMM904 model, (c) Metabolite abbreviations and names of fully coupled metabolite pairs for the AraCore model. (XLSX)

S3 Table Metabolites with the highest contribution in coupling relationships. (XLSX)

S4 Table Metabolite names and mutual pathway(s) of fully coupled pairs of iML1515. (XLSX)

S5 Table Metabolite names and mutual pathway(s) of fully coupled pairs of iMM904. (XLSX)

S6 Table Metabolite names and mutual pathway(s) of fully coupled pairs of AraCore. (XLSX)

S7 Table The Pearson correlations between the flux-sums and measured concentrations. (XLSX)
