# Supplementary material for: Flux-sum coupling analysis of metabolic network models
Source: PLoS Comput Biol. 2025 Apr 7;21(4):e1012972. doi: 10.1371/journal.pcbi.1012972 (PMC12005540; doi:10.1371/journal.pcbi.1012972)
Supplement: S1 Fig — Nodes represent metabolites, and edges depict the mutual pathways in which pairs of metabolites participate. Nodes with a degree smaller than 1 have been removed from the graph to improve visualization. Abbreviations for metabolites are used in the figure. The full names of the metabolites are given in Table B in S2 Table. (DOCX) [file pcbi.1012972.s001.docx]

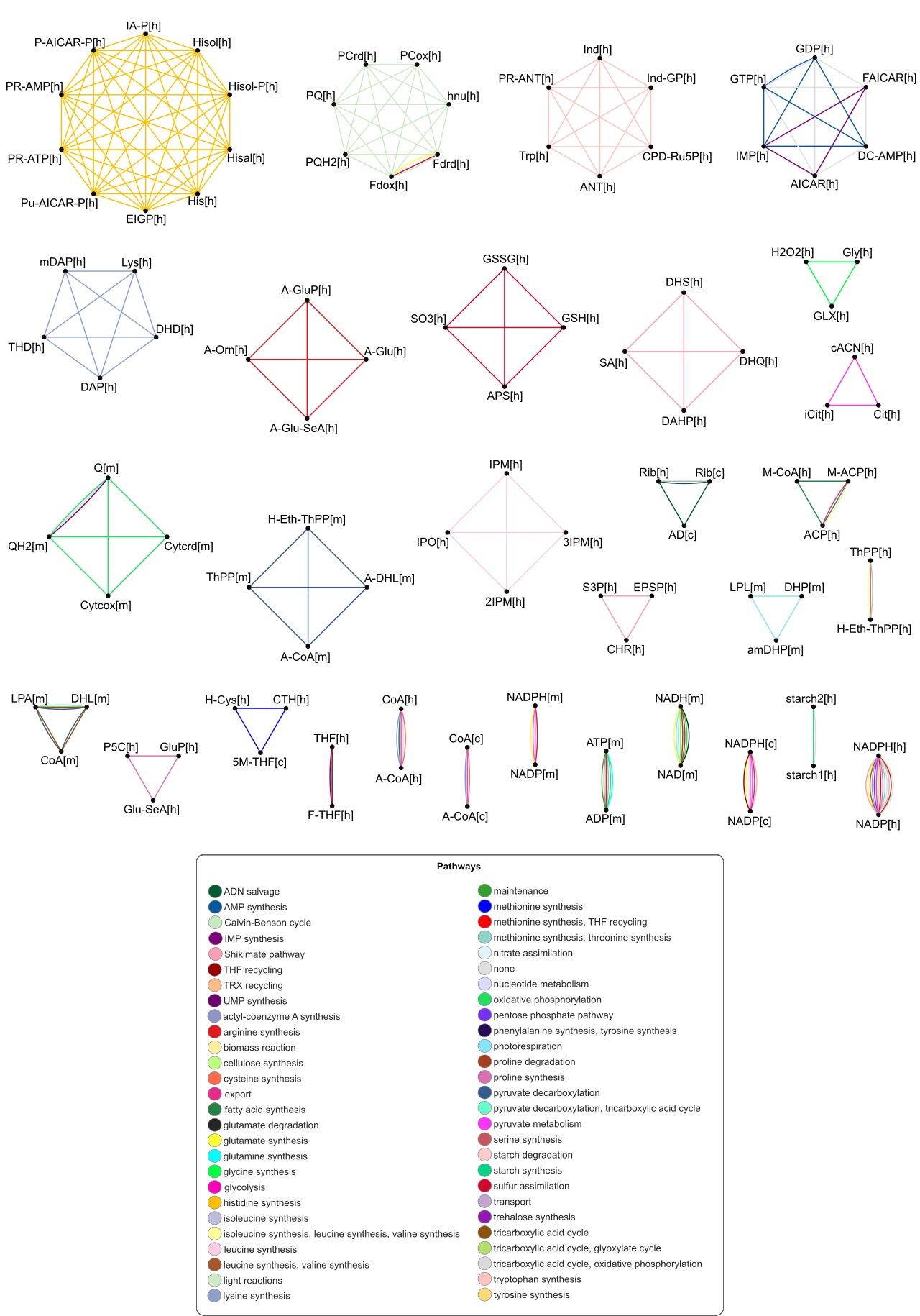


S1 Fig. Network representation of metabolites with fully coupled flux-sums in the iMM904 model. Nodes represent metabolites, and edges depict the mutual pathways in which pairs of metabolites participate. Nodes with a degree smaller than 1 have been removed from the graph to improve visualization. Abbreviations for metabolites are used in the figure. The full names of the metabolites are given in Table B in S2 Table.
