# Supplementary material for: Flux-sum coupling analysis of metabolic network models
Source: PLoS Comput Biol. 2025 Apr 7;21(4):e1012972. doi: 10.1371/journal.pcbi.1012972 (PMC12005540; doi:10.1371/journal.pcbi.1012972)
Supplement: S3 Fig — Scatter plots showing the flux sums of metabolites predicted by and relative metabolite concentrations over 27 conditions. The plots also include the Pearson correlation coefficients, along with p-values, and respective linear fits. (DOCX) [file pcbi.1012972.s003.docx]

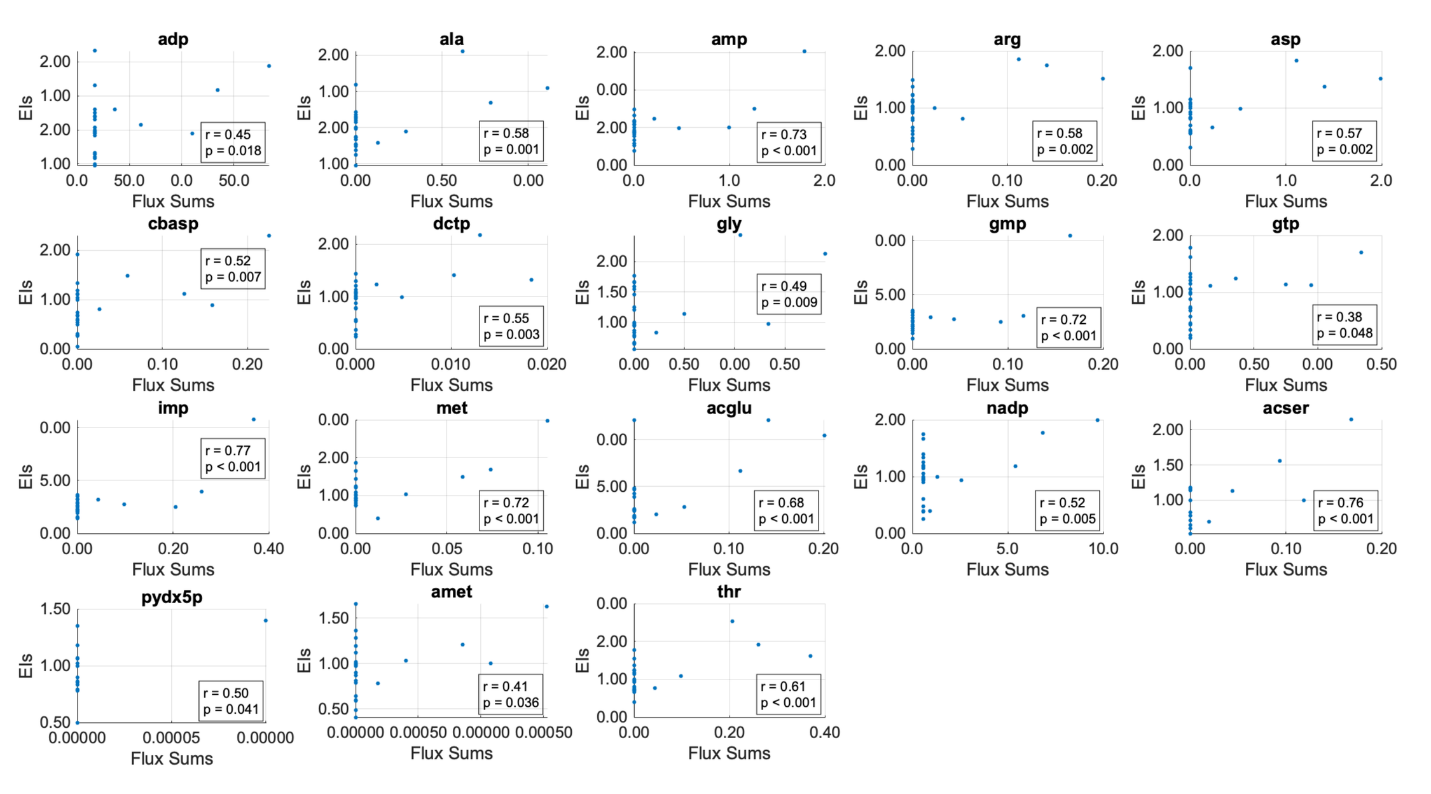


**S3 Fig.** **Scatterplots of correlation between estimated flux-sums and measured metabolite levels.** Scatter plots showing the flux sums of metabolites predicted by and relative metabolite concentrations over 27 conditions. The plots also include the Pearson correlation coefficients, along with p-values, and respective linear fits.
